# Scaffolded reaching experiences encourage grasping activity in infants at high risk for autism

**DOI:** 10.3389/fpsyg.2014.01071

**Published:** 2014-09-23

**Authors:** Klaus Libertus, Rebecca J. Landa

**Affiliations:** ^1^Learning Research and Development Center, University of PittsburghPittsburgh, PA, USA; ^2^Center for Autism and Related Disorders, Kennedy Krieger InstituteBaltimore, MD, USA

**Keywords:** infancy, motor development, grasping, sticky mittens, autism spectrum disorders

## Abstract

Recent findings suggest impaired motor skill development during infancy in children later diagnosed with autism spectrum disorders (ASD). However, it remains unclear whether infants at high familial risk for ASD would benefit from early interventions targeting the motor domain. The current study investigated this issue by providing 3-month-old infants at high familial risk for ASD with training experiences aimed at facilitating independent reaching. A group of 17 high-risk (HR) infants received 2 weeks of scaffolded reaching experiences using “sticky mittens,” and was compared to 72 low-risk (LR) infants experiencing the same or alternative training procedures. Results indicate that HR infants – just like LR infants – show an increase in grasping activity following “sticky mittens” training. In contrast to LR infants, evidence that motor training encouraged a preference for faces in HR infants was inconclusive.

## INTRODUCTION

Infants gather information about their environment and the objects within it through active exploration. The motor skills necessary to engage in manual exploration emerge during the first months of life as infants begin to reach for and grasp objects (e.g., White et al., 1964; Thelen et al., 1996). These basic exploration behaviors have far-reaching consequences for infants’ future development and research shows that early motor skills affects subsequent cognitive and social development. With regard to cognitive development, motor activity at age 2–5 months has been associated with attention skills at age 13 months (Tamis-Lemonda and Bornstein, 1993) and at age 8 years (Friedman et al., 2005). Similarly, motor maturity and exploration activity at age 5 months have been associated with academic achievement at age 14 years (Bornstein et al., 2013), and motor skills assessed via parent questionnaire between the ages of four to 48 months have been found to predict working memory and processing speed at school age (Piek et al., 2008). With regard to social development, motor and communicative skills have been found to correlate with each other (e.g., Hill, 2001). Motor skills at 18 months of age reportedly predict communication skills at 3 years of age (Wang et al., 2014), and the onset of independent walking has been found to increase both active social engagement by the child (Clearfield et al., 2008) and how mothers respond to social bids of the child (Karasik et al., 2014). Together, these examples highlight the importance of early motor skills for subsequent development and suggest that atypical motor skills may negatively impact development.

Additional evidence for the importance of early motor skills comes from children with developmental disorders. Motor delays are commonly reported in children with Down syndrome (Vicari, 2006), Williams syndrome (Masataka, 2001), in children born preterm (Caravale et al., 2005; van Haastert et al., 2006), and in children with an autism spectrum disorder (ASD; Ming et al., 2007). While these disorders are defined by specific cognitive, linguistic, or social delays, affected children also exhibit motor difficulties. With regard to ASD in particular, it has been suggested that motor delays during the first years of life may predict the social impairments that are characteristic of this disorder (Bhat et al., 2011). This hypothesis has been tested in infant siblings of children diagnosed with ASD who are at heightened risk to develop ASD or other developmental delays: approximately 20% of high-risk (HR) infants develop ASD themselves and another 30% show non-ASD developmental delays (Ozonoff et al., 2011). For example, Bhat et al. (2012) longitudinally examined motor development in 3- to 6-month-old HR infants and report that 70% of HR infants with early motor delays subsequently exhibited communication delays. Similarly, Flanagan et al. (2012) report that poor head control (i.e., head lag) in 6-month-old HR infants is associated with social delays, language impairments, and ASD at 36 months of age. Leonard et al. (2014) report that movement difficulties at age 9 months were associated with children’s ability to identify facial expressions and gaze direction at 5–7 years of age. And finally, LeBarton and Iverson (2013) report that fine motor skills in 12-month-old HR infant were a significant predictor of infants’ expressive language at 36 months of age. Together, these findings suggest that infants at HR for ASD show atypical motor development patterns that seem to predict outcomes in social and language domains. Interestingly, social deficits do not seem apparent during the first year of life in infants at risk or later diagnosed with ASD during what has been referred to as a “prodromal” period of the disorder (Elsabbagh and Johnson, 2010; Landa et al., 2013). For example, 7- and 14-month-old infants at HR for ASD seem similarly attracted to and interested in faces as their low risk (LR) peers (Elsabbagh et al., 2013). In contrast, poor motor skills have been reported in affected children already during early infancy and seem to be among the earliest signs of atypical developmental trajectories in HR infants (Bhat et al., 2011; Flanagan et al., 2012; Leonard and Hill, 2014).

Providing further evidence that critical motor skills may be affected early in ASD, a recent study reported reduced grasping engagement in 6-month-old HR infants (Libertus et al., 2014). Grasping is a foundational skill that opens up critical learning opportunities. Once an object has been grasped successfully, infants can learn about its function, use it for play, share it with others, or talk about it. These activities foster social and language skills. Consequently, reduced engagement in grasping behaviors may constrain opportunities for learning and contribute to poor developmental outcomes in some HR infants. Furthermore, motor delays seem to become more prominent over time in children later diagnosed with ASD (Landa et al., 2012; Lloyd et al., 2013) and may impact both children’ own exploratory behaviors and the verbal feedback they receive from their own caregivers (Karasik et al., 2014). Because of this “rate-limiting” role (Bushnell and Boudreau, 1993, p. 1017) of early motor skills, it seems logical to target the motor domain as part of early intervention strategies for HR infants. However, in reality the motor domain is rarely considered in ASD interventions, which focus primarily on communication and social interaction skills. The current study aims to fill this gap by investigating the effects of a low-cost, parent-implemented motor training paradigm in HR infants.

A number of studies have shown that typically developing 3-month-old infants (without a family history of ASD) respond to training that facilitates experiences of successful reaching using “sticky mittens.” For example, 2 weeks of parent-implemented training with “sticky mittens” have been found to encourage infants’ object exploration and grasping skills (Needham et al., 2002; Libertus and Needham, 2010). Other studies have reported changes in infants’ understanding and interpretation of another persons’ actions in familiar contexts following “sticky mittens” training (Sommerville et al., 2005; Skerry et al., 2013; Gerson and Woodward, 2014). And finally, Libertus and Needham (2011) examined whether successful grasping experiences influence infants’ preferences for faces and objects in a novel context. Their findings revealed, surprisingly, that experiences of successful grasping increased infants’ attention toward faces. A similar finding has since been obtained following experiences of independently moving an object that is attached to the child’s hand (Libertus and Needham, 2014). These studies demonstrate that scaffolded reaching experiences obtained via “sticky mittens” encourage both motor and social development in typically developing infants.

The current study builds upon these previous findings by investigating whether HR infants respond likewise to scaffolded reaching experiences (using “sticky mittens”). To this end, infants were assessed before, and after 2-weeks of “sticky mittens” training using a direct-observation measure of grasping behavior, a parent-report measure of early motor development, and an eye tracking measure of their preference for faces over toys. Results of the HR infants were compared to four LR groups receiving identical or different training procedures that were taken from previously published reports (Libertus and Needham, 2010, 2014). Results of the current study will inform early ASD intervention strategies by assessing whether HR infants respond to early motor training targeting grasping skills. Positive findings would encourage the implementation and assessment of motor-focused intervention in young children at risk for ASD (Lloyd et al., 2013).

## MATERIALS AND METHODS

The Institutional Review Board at Johns Hopkins University approved all study methods and materials. Parents of all participants provided written informed consent prior to their participation in this study.

### PARTICIPANTS

A total of 17 three-month-old infants participated in this experiment and completed 2 weeks of daily, parent-implemented training using “sticky mittens” (hereafter referred to as active training, or AT). All infants had an older sibling with a confirmed ASD diagnosis and were thus at heightened risk for ASD (HR infants). One infant was born premature at 35.5 weeks gestation; all others had full-term births (*M* = 38.72 weeks, SD = 1.29). An additional two infants were recruited but excluded from the final sample because they did not complete the full training protocol (*n* = 1) or returned late for their follow-up assessment (*n* = 1). Data from an additional 72 three-month-old infants without family history of ASD (LR infants) was obtained for comparison purposes from two previously published studies (Libertus and Needham, 2010, 2014). These additional LR infants provide critical comparison groups for the HR infants using the same and alternative training protocols (PT, passive training; EE, encouragement experience; or ME, movement experience). The five groups were of similar gender, and racial composition (see **Table 1** for details). There were no statistical differences between the groups with regard to age (*p*s > 0.339). Further, there were no statistical differences in training duration between the HR-AT and LR-AT or LR-PT groups (*p*s > 0.349). Training durations were significantly shorter in the LR-EE and LR-ME groups when compared to the HR-AT group (*p*s < 0.025).

**Table 1:** Participant characteristics.

Group	*N*	#*F*	Race	Age pre	TD	Age post
HR-AT	17	9	12C, 1B, 2A, 2M	11.04 (1.84)	135.31 (37.29)	13.06 (1.87)
LR-AT^a^	18	9	15C, 1A, 2M	10.90 (1.75)	125 (24.70)	12.92 (1.77)
LR-PT^a^	18	10	14C, 1B, 1A, 2M	10.90 (1.52)	144 (23.70)	12.93 (1.55)
LR-EE^b^	18	9	12C, 3B, 1A, 2M	10.51 (1.41)	109 (25.85)	13.09 (1.87)
LR-ME^b^	18	7	15C, 3B	10.87 (1.03)	101 (31.17)	13.33 (1.82)

*The total number of participants in each group (n) and the number of females per group (#*F*) are group totals. All other values are group averages with SD given in parentheses. TD, training duration (minutes). Age is reported in weeks. Race abbreviations: C, Caucasian, B, Black or African American, A, Asian, M, more than one race. Group abbreviations: HR, high-risk infants, LR, low-risk infants. Training abbreviations: AT, Active Training (“sticky mittens”), PT, Passive Training, EE, encouragement experience, ME, movement experience. Data from the gray shaded groups was previously reported in ^a^Libertus and Needham (2010) or ^b^Libertus and Needham (2014).*

### TRAINING

**FIGURE 1:**
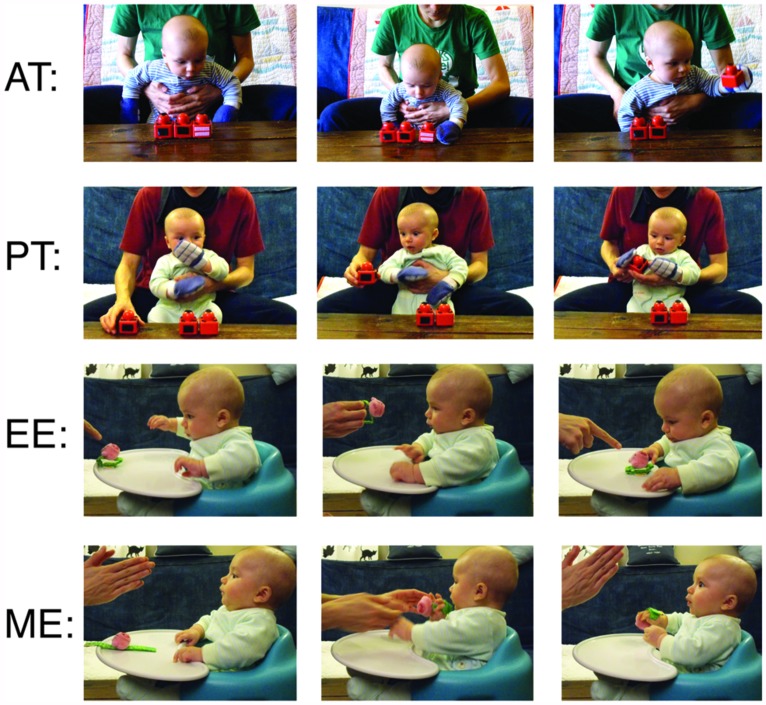
**Example of training procedures.** Active training (AT) – Infants wear “sticky mittens” allowing them to independently pick-up Velcro-covered toys. Passive training (PT) – Infants wear mittens without Velcro, parents lift and touch toy to infant’s palm. Encouragement experience (EE) – Parent draws attention to a small toy within reach of the infant, but does not help infant grasp toy. Movement experience (ME) – Parent engages infant but does not talk about or draw attention to toy, parent attached toy to infant’s hand without drawing attention to toy. Figure adapted from Libertus and Needham (2010, 2014).

Four training protocols were compared, each being administered by the infant’s own parent for 10 min each day for a duration of 2 weeks (for complete training details, please see Libertus and Needham, 2014). Training procedures are summarized in **Figure 1**.

#### Active training

Infants wore Velcro-covered mittens and Velcro-covered toys were placed in front of the child. Parents encouraged the child to reach for the toys. While wearing the mittens, accidental or purposeful contact with a toy made the toy stick to the mitten – providing experiences of successful object apprehension. Following successful toy contact, the child was allowed to explore the toy for approximately 10 s before the toy was removed and the procedure was repeated.

#### Passive training

Non-sticky mittens and toys, visually identical to the materials in the AT group were used. Parents placed the mittens over the child’s hands and touched the toys to their child’s palms/mittens, providing similar visual and tactile feedback as in the AT condition. Throughout the PT procedure, the child remained a passive observer and never independently moved or touched the toys themselves.

#### Encouragement experience

Parents placed a small, easily graspable toy in front of the child and drew attention to the toy by talking about it and pointing to it. Parents encouraged their child to reach for the toy, but were asked to refrain from helping their child obtain the object. Following successful toy contact, the child was allowed to explore the toy for approximately 10 s before the toy was removed and the procedure was repeated.

#### Movement experience

Parents first placed a small, easily graspable toy in front of the child and then attached it to the infant’s hand using Velcro straps. The toy was positioned so that its largest, most salient part was in the child’s palm. Throughout this procedure, the parent engaged the child in face-to-face interactions without mentioning or drawing attention to the toy. Consequently, infants experienced manual control over the object but no encouragement to act on it. Following 10 s of toy contact, the toy was removed and the procedure was repeated.

### PROCEDURE

All trained infants completed two visits to our lab, one before training (Pre) and one after 2 weeks of parent-implemented training (Post). Each lab visit was identical within and across groups and included a reaching assessment (Pre and Post) and a face preference assessment (Post only). HR infants receiving AT (HR-AT) and the LR infants in the EE (LR-EE) and ME (LR-ME) groups were tested twice, before and after training (for details see, Libertus and Needham, 2014). In contrast, LR infants in the AT (LR-AT) and PT (LR-PT) groups completed four additional reaching assessments in their own home during the training period (for details see, Libertus and Needham, 2010). Parents of infants in the HR-AT group additionally completed the Early Motor Questionnaire (EMQ; Libertus and Landa, 2013) before and after the 2 weeks of training.

### MEASURES

#### Reaching assessment

**FIGURE 2:**
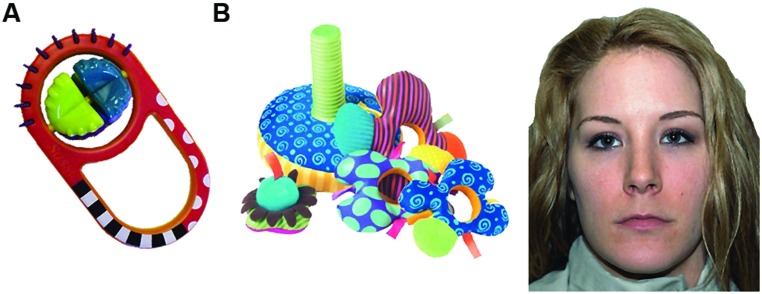
**Examples of toy used during reaching assessment (A), and of stimuli used during face preference task (B)**.

During each lab visit, infants completed a 1-min reaching assessment while sitting on their parent’s lap at a table. A small rattle toy (not used during training, see **Figure 2A**) was placed on the table in front of the child and the experimenter encouraged the child to reach for the toy. For the LR infants taken from previously published studies (Libertus and Needham, 2010, 2014), this task was split into two 30-s trials (within reach and next to hand) whereas the HR infants completed the task in one 60-s trial (within reach). Behavior was coded from video recordings by trained observers using frame-by-frame coding software. For the HR-AT group, two independent observers coded 15 randomly selected videos and correlation between the two observers was high (*r* = 0.90). Grasping behavior was defined as any manual contact with the toy that resulted in at least one corner of the toy being lifted off the table (as in Libertus and Needham, 2010).

#### Face preference task

Face preference was assessed using a paired visual comparison paradigm while infants’ eye gaze was recorded using a remote eye tracking system (Tobii 1750 for LR infants, Tobii X120 for HR infants). Infants were seated in a reclined infant seat or on their parent’s lap at a distance of approximately 60 cm from a 17″ screen (LR infants, 33.4 × 25.4° of visual angle) or approximately 75 cm from a 22″ screen (HR infants, 35.5 × 24.8° of visual angle). Four face-toy pairs were constructed from four realistic photographs of neutral faces (two female, all Caucasian) and four photographs of infant toys. Faces were selected from the NimStim stimulus set (Tottenham et al., 2009), toys were commercially available infant toys not used during training or during the reaching assessment. Faces and Toys were similar in size and luminance (DeNicola et al., 2013) and were presented simultaneously, side-by-side (see **Figure 2B**). Face preference scores were defined as in previous studies as the difference between infants’ proportion of looking to the face minus looking to the toy (with %LT face + %LT toy = 100%).

#### Early Motor Questionnaire

The EMQ is a parent-report measure of motor development designed for children ranging from 2 to 24 months of age. The EMQ consists of three separate sections assessing gross motor skills (GM: 49 items), fine motor skills (FM: 48 items), and perception-action skills (PA: 31 items). High concurrent and predictive validity of EMQ scores with corresponding measures on the established Mullen Scales of Early Learning (MSELl; Mullen, 1995) and Peabody Developmental Motor Scales, second edition (PDMS-2; Folio and Fewell, 2000) have been reported in a sample that included children at HR for ASD (Libertus and Landa, 2013). The EMQ is currently the only motor development questionnaire suitable for 3-month-olds that has been compared to standardized assessment and that has included HR infants in these comparisons. Parents of HR infants were asked to complete the EMQ the day before their visit to our lab. If a parent failed to complete the EMQ prior to their lab visit, they either completed the EMQ during their lab visit or later the same day. EMQ raw scores have different ranges for the three sub-scales, range from -98 to +98 in the GM domain, -96 to +96 in the FM domain, and -62 to +62 in the PA domain. For display purposes, EMQ raw scores were adjusted by adding 75 to GM and FM scores, and by adding 35 to PA scores.

### ANALYSIS

Differences in grasping duration were examined using analysis of variance (ANOVA) prior to training and using analysis of covariance (ANCOVA, including pre-training grasping duration as covariate) following 2 weeks of training with Group (5) as between-subjects factor. The ANCOVA was followed by planned comparisons between the HR-AT group and all other groups in the model. Finally, within-group analyses were conducted using paired *t*-tests comparing pre and post training scores on the EMQ and reaching assessment, and using one-sample *t*-tests to assess face preference following training. Where applicable, partial eta-squared ($\eta_{p}^{2}$) or Cohen’s *d* was calculated as measures of effect size.

## RESULTS

Preliminary analyses revealed no effects of Gender on infants’ grasping behavior, EMQ scores, or face preference scores (*p*s > 0.220). Therefore, Gender was excluded from all subsequent analyses.

### REACHING ASSESSMENT

#### Between-group analyses

A one-way ANOVA revealed no differences in baseline grasping duration between any of the groups prior to training (*p* = 0.715). Following 2 weeks of parent-implemented training, an analysis of change using ANCOVA (with pre-training grasping duration as covariate) revealed a significant main effect of Group, *F*(4,83) = 3.068, *p* = 0.021, $\eta_{p}^{2}$ = 0.129. Planned comparisons revealed significantly longer grasping durations following training in the HR-AT group (*M* = 31.37, SD = 26.21) than in the LR-PT group [*M* = 11.80, SD = 14.70; *p* = 0.018, *95% CI* (-31.04, -3.06)], the LR-EE group [*M* = 16.07, SD = 17.74; *p* = 0.035, *95% CI* (-28.86, -1.11)], or the LR-ME group [*M* = 13.10, SD = 18.53; *p* = 0.018, *95% CI* (-31.04, -3.06)]. In contrast, there were no significant differences between the HR-AT and the LR-AT group (*M* = 29.93, SD = 16.82; *p* = 0.901).

#### Within-group analyses

**FIGURE 3:**
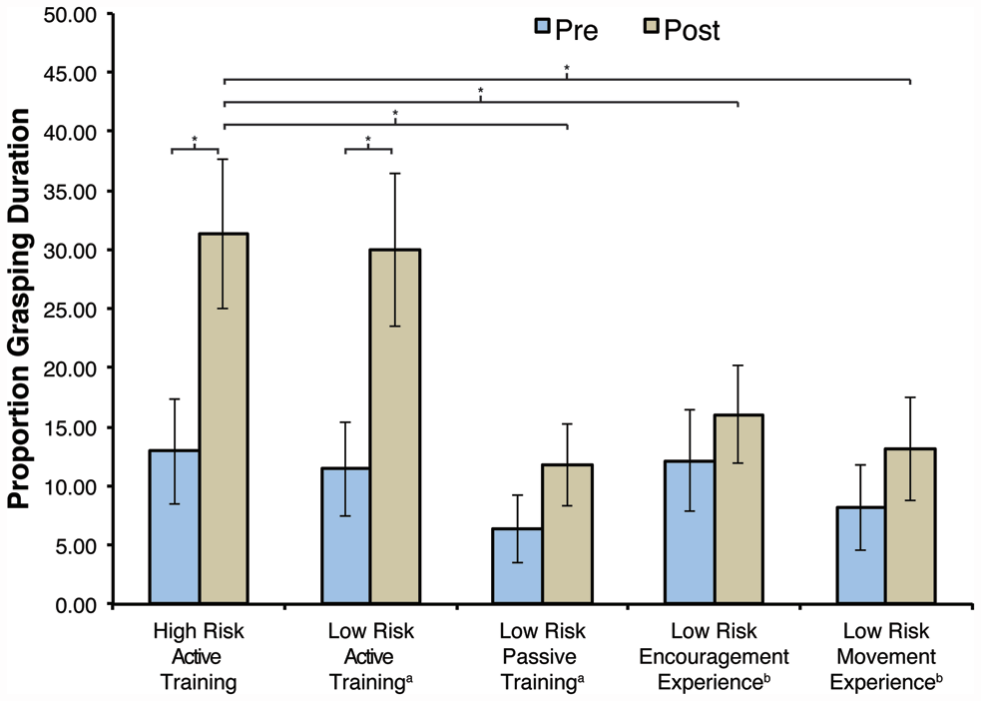
**Average grasping duration before (Pre) and after (Post) training by group.** Data from the low risk (LR) groups was taken from (a) Libertus and Needham (2010) or (b) Libertus and Needham (2014). Error bars represent SEM, *p < 0.05.

Paired-sample *t*-tests were used to compare grasping duration before and after training within each group. Results revealed a significant increase in grasping duration in the HR-AT group, *t*(16) = 3.084, *p* = 0.007, *d* = 0.82, and the LR-AT group, *t*(17) = 3.857, *p* = 0.001, *d* = 0.81. No significant changes in grasping duration were observed in the remaining groups (*p*s > 0.265). These findings are summarized in **Figure 3**.

### EARLY MOTOR QUESTIONNAIRE

**FIGURE 4:**
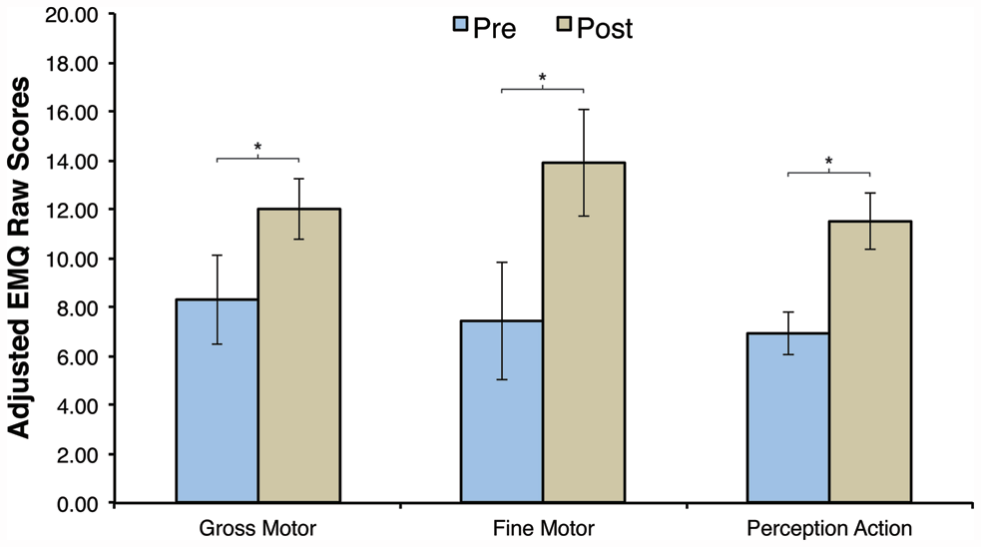
**Adjusted Early Motor Questionnaire (EMQ) raw scores before (Pre) and after (Post) training for the high risk active training group.** Error bars are SEM, *p < 0.05.

The current study was the first to include a parent-report measure to assess the effect of AT on motor skill development. Consequently, EMQ scores were only available for the HR-AT group. Paired-sample *t*-tests revealed significant increases in EMQ raw scores in the GM domain (Pre: *M* = -66.69, SD = 7.28; Post: *M* = -63.00, SD = 5.02), *t*(15) = 2.357, *p* = 0.032, *d* = 0.59, the FM domain (Pre: *M* = -67.56, SD = 9.65; Post: *M* = -61.13, SD = 8.70), *t*(15) = 2.565, *p* = 0.022, *d* = 0.70, and in the perception-action domain (Pre: *M* = -28.06, SD = 3.49; Post: *M* = -23.50, SD = 4.53), *t*(15) = 3.607, *p* = 0.003, *d* = 1.13. These findings parallel the observed increases in grasping duration reported above. However, it is important to note that parents’ interactions with their child during the AT may have affected their EMQ ratings. The EMQ results are summarized in **Figure 4**.

### FACE PREFERENCE TASK

**FIGURE 5:**
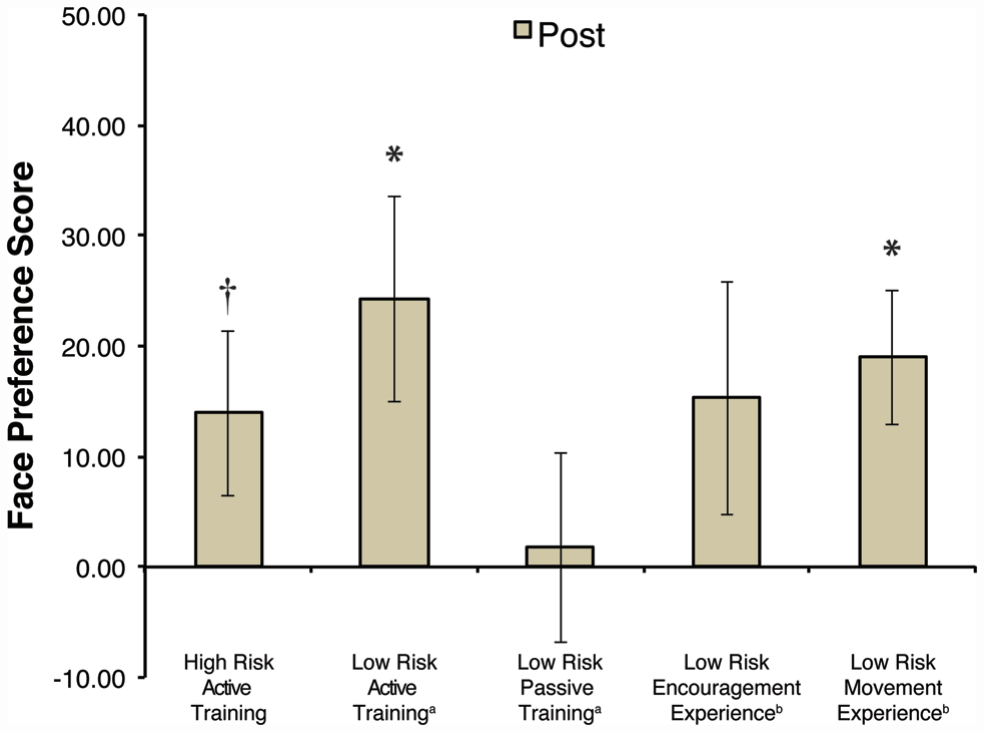
**Average face preference scores after training (Post) by group.** Data from the LR groups was taken from (a) Libertus and Needham (2011) or (b) Libertus and Needham (2014). Error bars represent SEM, *p < 0.05, ^†^*p* = 0.086.

Previously published findings (Libertus and Needham, 2014) have noted a significant preference for faces over toys in the LR-AT group (*M* = 24.24, SD = 38.32), *t*(16) = 2.609, *p* = 0.019 and in the LR-ME group (*M* = 19.01, SD = 25.20), *t*(16) = 3.110, *p* = 0.007. In contrast, no face preference was observed in the LR-EE group (*M* = 15.27, SD = 43.42; *p* = 0.166) or the LR-PT group (*M* = 1.78, SD = 36.46; *p* = 0.839). Using the same task, the HR-AT group showed an overall positive preference for faces but this effect failed to reach statistical significance (*M* = 13.92, SD = 29.16; *t*(14) = 1.849, *p* = 0.086; see **Figure 5**). This result needs to be interpreted with caution, as stimuli were presented on different sized screens (17″ vs. 22″) between HR and LR infants. However, the actual area taken up by the face-toy pairs in the visual field was similar across studies (see Materials and Methods).

### RELATION BETWEEN FACE PREFERENCE AND GRASPING ACTIVITY

The relation between infants’ preference for faces and their grasping behavior was assessed using Pearson’s correlation by comparing infants’ proportion of grasping duration after training (reported in **Figure 3**) to their face preference scores after training (reported in **Figure 5**). Due to the small sample sizes within each group, correlations were not statistically significant for individual groups and need to be interpreted with caution. Nevertheless, the three LR groups that have obtained some object manipulation experiences during training (AT, EE, and ME groups) showed similar positive correlations between face preference and grasping duration scores (LR-AT: *r* = 0.315, *p* = 0.218; LR-ME: *r* = 0.363, *p* = 0.152; LR-EE: *r* = 0.387, *p* = 0.125). Combining these three groups resulted in a significant positive correlation of comparable magnitude as in the individual groups (*r* = 0.344, *p* = 0.013). In contrast, the LR-PT group exhibited no correlation between face preference and grasping activity (LR-PT: *r* = -0.059, *p* = 0.816). Interestingly, the HR-AT group also showed no correlation between face preference and grasping activity (*r* = 0.016, *p* = 0.955), exhibiting a pattern closer to the LR-PT group than to the LR-AT group. This pattern could suggest that the connection between early motor experiences and face preference may be disrupted in at least some HR infants. This intriguing preliminary observation should be investigated in more detail in the future.

## DISCUSSION

The current study investigated the response of infants at high familial risk (HR) for ASD to parent-implemented motor training. Our results provide evidence that 3-month-old HR infants are able to learn from scaffolded reaching experiences (AT using “sticky mittens”) and are likely to respond to treatment interventions targeting the motor domain. In fact, behavior of the HR infants assessed here paralleled that of LR infants reported in Libertus and Needham (2010). Both LR and HR infants showed similar levels of grasping activity before (*M*_HR_ = 12.93; *M*_LR_ = 11.43) and following 2-weeks of AT (*M*_HR_ = 31.37; *M*_LR_ = 29.93). In addition, the current study used a complementary parent-report measure (EMQ) and revealed increases in GM, FM, and perception-action integration skills following training in the HR group. These results are the first to show that HR infants respond to parent-implemented motor training using “sticky mittens” and that that the effects of such training are evident via parent-report. Finally, the current study also investigated infants’ preference for faces over toys following 2 weeks of motor training. In contrast to the LR infants, the HR group did not show a clear preference for faces over toys following training. This result is surprising and may suggest differences in learning from motor experiences in some HR infants – although future research on this issue is needed.

### MOTOR DEVELOPMENT IN ASD

Our preliminary findings show that 3-month-old HR infants respond well to an intervention targeting their early motor skills, but should not be interpreted as providing an early ASD treatment. Rather, our findings are a first step that should encourage future research toward the development of ASD intervention paradigms incorporating the motor domain. A number of studies have suggested that infants later diagnosed with ASD or who are at high familial risk for ASD show atypical motor development in infancy. For example, prospective studies with HR infants have shown subtle motor delays that persist or become more prominent as children grow older (e.g., Landa et al., 2012; Leonard et al., 2013; Lloyd et al., 2013). Further, motor delays are more common in HR than in LR infants and poor postural control skills have been observed in HR infants (Bhat et al., 2011; Nickel et al., 2013). More recently, reduced grasping activity and poor grasping-related fine motor skills have been reported in 6-month-old HR infants (Libertus et al., 2014). Nevertheless, it remains unclear whether delayed or atypical motor skills are related to subsequent ASD symptoms. Such a connection between motor and social skills has been theorized in ASD (Bhat et al., 2011), and empirical evidence suggests that early motor skills may affect subsequent development of social communication skills and may even predict ASD in HR infants (Bhat et al., 2012; Flanagan et al., 2012; LeBarton and Iverson, 2013; Leonard et al., 2014). These findings suggest that atypical or delayed motor skills in early infancy may contribute to (*not cause*) subsequent ASD symptoms and should be targeted in early intervention efforts. Our current findings indicate that HR infants may indeed respond to early motor training targeting their grasping skills, which have been identified as an area of weakness in 6-month-old HR infants (Libertus et al., 2014). Longitudinal follow-up studies are now necessary to determine whether such early motor training may also facilitate subsequent social or language development in HR infants.

### INFLUENCES OF MOTOR SKILLS FOR SOCIAL DEVELOPMENT

Motor development can affect subsequent development in seemingly unrelated domains by providing infants and toddlers with the necessary means to engage with the world around them. For example, studies have shown that motor experiences and first-hand opportunities to explore objects impact infants’ perceptual development (e.g., Gibson and Pick, 2000; Bourgeois et al., 2005; Soska et al., 2010; Schwarzer et al., 2012). However, motor experiences are also critical for social interactions and exchanges – a relation that is often overlooked. For instance, grasping allows infants to show or share objects with others, opening up opportunities for social exchanges, joint attention, and learning. A recent review of 43 studies on the relation between motor and social skills confirms the importance of early motor skills for infants’ social development (Leonard and Hill, 2014).

Direct evidence for a relation between motor experiences and social development comes from studies using “sticky mittens” to provide 3-month-old infants’ who are not reaching on their own yet with experiences of successful reaching. Using this paradigm, studies have reported changes in infants’ understanding and interpretation of another persons’ actions and in their preference for faces (Sommerville et al., 2005; Libertus and Needham, 2011; Skerry et al., 2013; Gerson and Woodward, 2014). More recently, significant correlations between infants’ motor activity and their preference for faces have been reported in untrained, naïve infants (Libertus and Needham, in press). Together, these studies suggest that experiences of obtaining and manipulating objects encourage social attention in typically developing 3-month-old infants. Using the same paradigm, the current study provides only limited evidence for a relation between motor experiences and face preference in HR infants. Previous work suggests that the development of face preference and processing skills may be altered in HR infants. For example, infants later diagnosed with ASD seem to reduce their attention toward the eye region in faces between 2 and 6 month of age (Jones and Klin, 2013). Similarly, 6-month-olds later diagnosed with ASD seem less interested in faces within the context of complex visual scenes than typically developing infants (Chawarska et al., 2013). But at the same time, 7-month-old HR infants seem to show a stronger orienting response and preference for faces than LR peers within the context of simple visual displays (Elsabbagh et al., 2013). Together, these findings suggest that face preference and processing skills are not simply absent in HR infants, but seem to follow a different developmental trajectory. Our findings on the face preference task may reflect such differences in face processing skills in HR infants and could be driven by overall more variability within this group (reducing statistical power) or by a sub-set of HR infants who will eventually be diagnosed with ASD. Follow-up assessments of the HR infants reported here are planned to investigate this possibility.

### LIMITATIONS

The findings reported here are interesting and may inform the design and implementation of future ASD intervention paradigms. Nonetheless, there are limitations that need to be considered. First of all, no untrained or PT-HR control group was included in the current sample. Given the overall similar performance between the HR-AT and LR-AT groups, we would not expect a HR-PT group to perform differently from the LR-PT group on the reaching assessment or face preference task. However, having a HR-PT group would be of interest for interpreting EMQ results. While in agreement with our direct-observation measures, the impact of parental bias on EMQ scores remains unknown. Further, it is unclear whether alternate training procedures such as PT would also affect EMQ scores. For example, it is possible that the increases in EMQ GM scores stem from additional sitting experiences provided during the training. These experiences are shared between the AT and PT groups and consequently EMQ GM scores might increase following either AT or PT procedures.

Further limitations of the current study are the slight methodological differences between the HR and LR groups. For example, the reaching assessment was administered in a slightly different way to the LR groups than to the HR groups, and stimuli for the face preference task were presented on different sized screens between the HR and LR groups (see Materials and Methods). The fact that we obtained highly similar results in the HR-AT and LR-AT groups suggests that these differences did not influence our results.

## CONCLUSION

In conclusion, our findings suggest that 3-month-old infants at HR for ASD respond to early motor-focused interventions. Following 2 weeks of parent-implemented training using “sticky mittens,” HR infants showed increased grasping activity during object exploration and scored higher on a parent-report measure of early motor skills. These results replicate previous findings obtained with LR infants and extend them to a population known to exhibit reduced grasping and motor skills in infancy. At the same time, increased grasping experiences during and following training did not seem to stimulate attention toward faces in HR infants. Although the motor training was not set-up to encourage a preference for faces, this result stands in contrast to prior findings with LR infants. It is possible that the motor and social skills of some HR infants are not connected in the same way as in LR infants during the ages assessed here (3-month-olds). Longitudinal follow-up investigations will be conducted to examine this possibility further.

## Conflict of Interest Statement

The authors declare that the research was conducted in the absence of any commercial or financial relationships that could be construed as a potential conflict of interest.
